# ^18^F-FDG-PET uptake in non-infected total hip prostheses

**DOI:** 10.1080/17453674.2018.1525931

**Published:** 2018-10-18

**Authors:** Stefan J Gelderman, Paul C Jutte, Ronald Boellaard, Joris J W Ploegmakers, David Vállez García, Greetje A Kampinga, Andor W J M Glaudemans, Marjan Wouthuyzen-Bakker

**Affiliations:** 1 Medical Imaging Center, Department of Nuclear Medicine and Molecular Imaging, University of Groningen, University Medical Center Groningen, Groningen;;; 2 Department of Orthopaedic Surgery, University of Groningen, University Medical Center Groningen, Groningen;;; 3 Department of Medical Microbiology and Infection Prevention, University of Groningen, University Medical Center Groningen, Groningen, the Netherlands

## Abstract

Background and purpose — ^18^F-fluorodeoxyglucose positron emission tomography (FDG-PET) can be used in the diagnostic work-up of a patient with suspected periprosthetic joint infection (PJI) but, due to a lack of accurate interpretation criteria, this technique is not routinely applied. Since the physiological uptake pattern of FDG around a joint prosthesis is not fully elucidated, we determined the physiological FDG uptake in non-infected total hip prostheses.

Patients and methods — Patients treated with primary total hip arthroplasty (1995–2016) who underwent a FDG-PET/CT for an indication other than a suspected PJI were retrospectively evaluated. Scans were both visually and quantitatively analyzed. Semi-quantitative analysis was performed by calculating maximum and peak standardized uptake values (SUV_max_ and SUV_peak_) by volume of interests (VOIs) at 8 different locations around the prosthesis.

Results — 58 scans from 30 patients were analyzed. In most hips, a diffuse heterogeneous uptake pattern around the prosthesis was observed (in 32/38 of the cemented prostheses, and in 16/20 of the uncemented prostheses) and most uptake was located around the neck of the prosthesis. The median SUV_max_ in the cemented group was 2.66 (95% CI 2.51–3.10) and in the uncemented group 2.87 (CI 2.65–4.63) (Median difference = –0.36 [CI –1.2 to 0.34]). In uncemented prostheses, there was a positive correlation in time between the age of the prosthesis and the FDG uptake (r_s_ = 0.63 [CI 0.26–0.84]).

Interpretation — Our study provides key data to develop accurate interpretation criteria to differentiate between physiological uptake and infection in patients with a prosthetic joint.

Diagnosing a periprosthetic joint infection (PJI) can be a clinical challenge, especially for chronic and low-grade infections. The preoperative diagnostic work-up, e.g. serum C-reactive protein (CRP), erythrocyte sedimentation rate (ESR), synovial culture, and several synovial biomarkers can be false negative or false positive (Hozack et al. 1991, Ivanèeviae et al. 2002, Koh et al. 2017). The currently applied diagnostic criteria as described by the Musculoskeletal Infection Society (MSIS) to diagnose a PJI do not entail advanced nuclear imaging –(Parvizi et al. 2011). However, given the difficulty of the diagnosis of PJI and the rapidly evolving field of nuclear diagnostics, it may be beneficial to add this technology to the diagnostic possibilities.

Several nuclear medicine imaging techniques are available to assist in diagnosing or excluding PJI, but the choice of which technique to use first depends mostly on local expertise, availability of techniques, and costs. The labelled white blood cell (WBC) scintigraphy is currently considered as the gold standard, because of its high specificity for infection (Jutte et al. 2014) but has its limitations. The technique is time-consuming for both patient and personnel, is not available in all hospitals, and patients with leukopenia are not suitable for the test due to a low labelling efficacy (Glaudemans et al. 2013, Palestro 2015).

A good alternative for WBC scintigraphy could be ^18^F-fluorodeoxyglucose positron emission tomography (FDG-PET), which can be combined with computed tomography (CT) for exact anatomical localization (the CT part) of the accumulation of FDG (Boellaard et al. 2015). FDG-PET is widely available, more comfortable for the patient, provides better possibilities for quantification, and results in higher resolution images compared with WBC scintigraphy (Love et al. 2005, Verberne et al. 2016). However, an important limitation of FDG-PET/CT when compared with WBC scintigraphy is its lack of discrimination between infection and inflammation secondary to a physiological reaction to the foreign body (the implant). In addition, it is not known how long physiological FDG uptake around a prosthesis remains after primary arthroplasty (Delank et al. 2006, Glaudemans et al. 2013). Consequently, diagnostic interpretation criteria for declaring an FDG-PET/CT positive or negative for PJI are lacking and, as a consequence, FDG-PET/CT is not routinely performed for the diagnosis of PJI. Therefore, we determined the physiological FDG uptake in non-infected total hip prostheses.

## Patients and methods

### Patients

Data of patients who underwent a primary total hip arthroplasty between January 1995 and December 2016 and underwent an FDG-PET/CT scan in the time period after the primary arthroplasty for reasons other than a suspected PJI (mostly for oncological reasons) were collected retrospectively. Patients were included only if they had no clinical signs (e.g., pain at rest) of a PJI at the time the FDG-PET/CT scan was performed. The included patients were divided into 2 groups, cemented and uncemented.

### FDG-PET/CT imaging

All FDG-PET/CT scans were performed with an EARL accredited scanner, Siemens Biograph MCT 64 or 40 slice (Knoxville, TN, USA). Patients were instructed to fast for at least 6 hours prior to the administration of FDG. Blood glucose levels were measured before injection of the FDG and had to be below 11 mmol/L. The acquisition of the scan started after a resting time of approximately 60 minutes after 3 MBq/kg FDG administration. All scan acquisition parameters were according to existing guidelines from the European Association of Nuclear Medicine (EANM) (Boellaard et al. 2015). All analyses were performed on EARL reconstructed images.

### Image interpretation and analysis

Both visual and semi-quantitative analysis was performed to evaluate the FDG uptake surrounding the prosthesis. For the visual analysis, FDG uptake was categorized in 4 categories: diffuse heterogeneous (uptake around the prosthesis with different intensities), diffuse homogeneous (relative constant uptake around the prosthesis), focal (1 spot with high intensity), and no uptake. In addition, when there was extension of FDG uptake in the soft tissues surrounding the prosthesis this was recorded.

For the semi-quantitative analysis, volumes of interests (VOIs) were created in each scan at 8 different locations around the prosthesis (tip of the prosthesis, femur–prosthesis interface, greater and lesser trochanter, neck of the prosthesis, lateral and medial acetabulum, and interface between the prosthesis and the median part of the acetabulum). From these VOIs the maximum and peak standardized uptake value (SUV max and SUVpeak, which are values for the amount of FDG uptake) were determined.

The mean of the SUV_max_ and SUV_peak_ at the 8 regions was calculated, as a representative of the uptake of the whole prosthesis (meanSUV_max_ and meanSUV_peak_). These values were corrected for background SUV, using the mean SUV (SUV_mean_) of the liver (meanSUV_max_/liver ratio and meanSUV_peak_/liver ratio). Finally, the correlation of the 4 variables between the age of the prosthesis and FDG uptake was determined.

### Statistics

IBM SPSS statistics version 20 (IBM Corp, Armonk, NY, USA) was used for statistical analysis. Additionally, StatsDirect version 3 (https://www.statsdirect.co.uk/) was used for calculating the confidence intervals (CI) for both the difference in mean values and the correlation coefficient. Since in clinical practice a PJI can occur in any joint prosthesis, regardless of the age of the prosthesis, all values were considered practically relevant and included in the CI. Continuous variables were presented as means (SD) or (95% confidence intervals—CI). Not normally distributed data were presented with the median and interquartile ranges (IQR) or with the CI of the mean. Differences between groups were calculated with the Mann–Whitney U-test. Correlation between the age of the prosthesis and the SUV uptake was calculated with Spearman’s rho. Differences in uptake between the locations were calculated with Friedman’s ANOVA. In the case of a statistically significant result, Wilcoxon’s signed rank test was used for post hoc calculation. A p-value <0.05 was considered significant, without correction for multiple comparisons. Effect size estimates (r) for Mann–Whitney U and Wilcoxon signed rank test was calculated as *r* = *z ⁄* √*N* (Fritz et al. 2012), with *z* being the standardized test statistics and N the sample size. Effect sizes were interpreted as large (> 0.5), medium (0.3–0.5), and small (< 0.3). Moreover, to overcome the problem of correlated observations, additional analyses were done with only the last available scan of each patient.

### Ethics, funding, and potential conflicts of interest

Ethical permission was acquired for this study from the UMCG medical ethical committee on 21 November 2017 (number M17.221053). None of the authors has had funding that might pose a conflict of interest in connection with the submitted article.

## Results

### Patients

**Figure 1.**
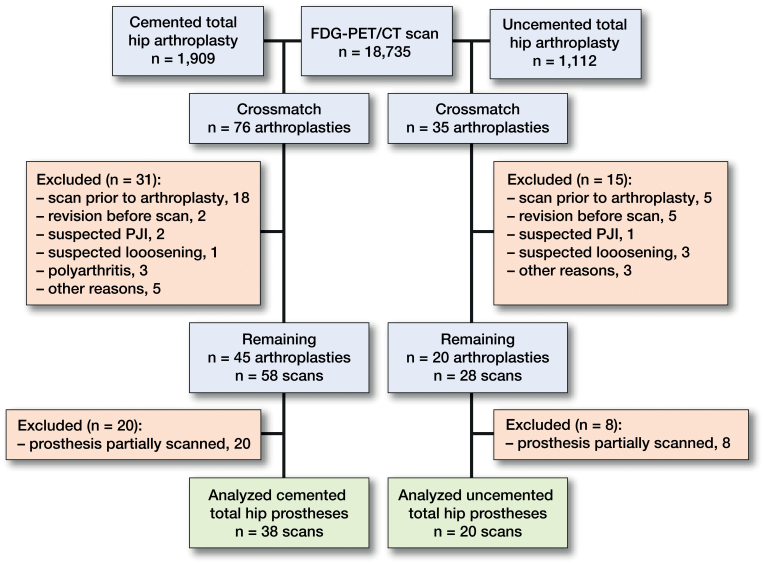
Flow chart of the patient inclusion process.

Finally, 33 patients, with 42 total hip prostheses and 58 FDG-PET/CT scans, were included (Figure 1). The indication for 37 FDG-PET/CT scans was because of a malignancy, the commonest being lung carcinoma. In the non-oncological group, scans were performed in patients with increased inflammatory parameters (n = 8) or other indications (n = 4). Similar FDG uptake was found between oncological (meanSUV_max_ = 2.57 [IQR 2.08–3.21]) and non-oncological indicated scans (meanSUV_max_ = 3.01 [IQR 2.32–3.95]).

The cemented group consisted of 38 prostheses scanned in 21 patients (median age 73 years [IQR 66–79]). 19 of these prostheses were scanned once in 15 different patients. 7 cemented hip prostheses in 5 patients were scanned twice, with a median time between the 2 scans of 1 year (IQR 0.4–3.9). 1 hip prosthesis was scanned 5 times within a duration of approximately 4 years. The uncemented group consisted of 20 scans from 12 patients (median age 52 years [IQR 50–67]). 9 patients (11 prostheses) were scanned once. 2 patients with 3 prostheses were scanned twice (median time between 2 scans: 0.2 years [IQR 0.1–0.2]), and 1 hip prosthesis in a different patient was scanned 3 times within a duration of 9 months.

### Visual analysis

**Figure 2.**
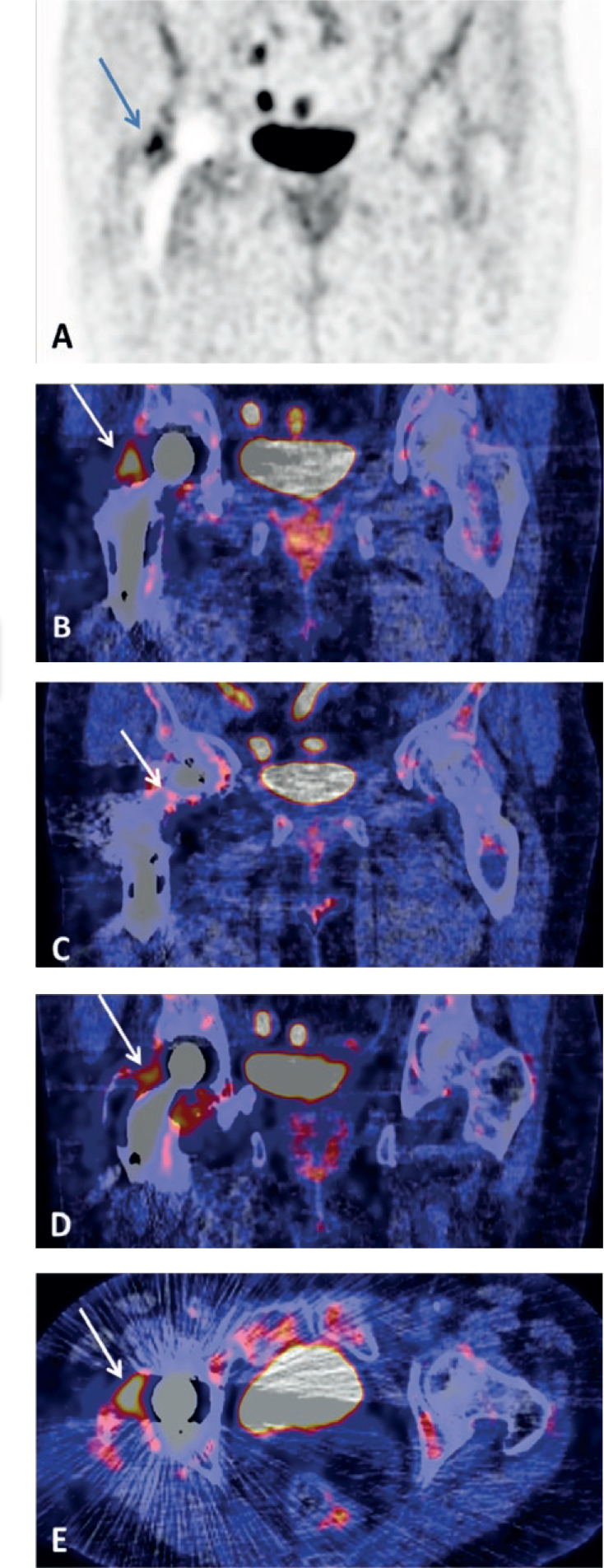
Example of ^18^F-FDG uptake in a non-infected total hip prosthesis. (A) Coronal FDG-PET image showing FDG uptake around the prosthesis, most prominent at the lateral side of the collum; (B, C, D) Coronal fused FDG-PET/CT images at different slices showing the uptake around the prosthesis; (E) Transaxial fused FDG-PET/CT image showing prominent physiological uptake at the lateral side of the cup of the prosthesis.

Visually, 39/42 prostheses showed uptake of FDG around the prosthesis. Diffuse heterogeneous uptake (Figure 2) was the most common uptake pattern in both the cemented group (32/38) and the uncemented group (16/20) (Table 1). No FDG uptake was seen in the periprosthetic soft tissues surrounding the prosthesis, except for the soft tissue at the height of the greater trochanter.

**Table 1.** Visual FDG-uptake pattern in non-infected hip prostheses

	Cemented	Uncemented
Factor	n = 38	n = 20
Diffuse heterogeneous	32	16
Diffuse homogeneous	2	2
Focal	2	0
No uptake	2	2

### Semi-quantitative analysis

The median value of the meanSUV_max_ in the cemented group was 2.7 (CI 2.5–3.1) and in the uncemented group 2.9 (CI 2.7–4.6) (median difference –0.36 [CI –1.2 to 0.34]). Also, without correlated observations the groups were similar. Other variables did not differ statistically significantly between groups either: meanSUV_peak_ (*r* = –0.10, p = 0.4), meanSUV_max_/liver ratio (*r* = –0.25, p = 0.06), meanSUV_peak_/liver ratio (*r* = –0.26, p = 0.05). The background uptake (SUV_mean_) in the liver was for the cemented group 2.9 (IQR 2.6–3.3), for the uncemented group 2.7 (IQR 2.3–3.0). The age of the patient did not statistically significantly influence the FDG uptake in either the cemented group (*r_s_* = 0.20, p = 0.2) or the uncemented group (*r_s_* = 0.10, p = 0.7). The SUV_max_ at the 8 different locations around the prosthesis is given in Table 2. The highest uptake was observed around the neck of the prosthesis and was significantly different compared with the other locations in both the cemented (*r* = [range –0.57 to –0.87], p < 0.001) and the uncemented group (*r* = [range –0.74 to –0.88], p < 0.001). Furthermore, other locations with increased uptake were observed at the lateral and medial acetabulum and the greater and lesser trochanter. The tip of the prosthesis showed minimal uptake. In addition, minimal uptake was observed at the femur–prosthesis interface of uncemented hip prostheses. FDG uptake in this location was absent in 34/38 cemented hip prostheses. The uptake between the cemented and uncemented prostheses was significantly different at the femur–prosthesis interface location (*r* = –0.37, p = 0.01) (*r* = –0.29, p = 0.06 without correlated observations). FDG uptake at other locations was similar between the cemented and uncemented prostheses.

**Table 2.** SUVmax measured at 8 different locations around the prosthesis

	Cemented group	Uncemented group
	Median SUV_max_ (IQR)	Median SUV_max_ (IQR)
Tip	2.0 (1.6–2.4)	2.2 (1.1–3.1)
Shaft	0.8 (0.6–1.1)	1.1 (0.9–2.0)
Greater trochanter	3.3 (2.5–4.0)	2.2 (1.7–4.2)
Lesser trochanter	2.8 (2.3–3.5)	2.6 (1.7–4.2)
Neck	4.2 (2.9–5.4)	4.6 (3.1–10.4)
Acetabulum lateral	3.1 (2.5–4.4)	3.2 (2.4–6.3)
Acetabulum median	2.0 (1.6–2.6)	1.9 (1.4–3.4)
Acetabulum medial	2.7 (2.0–3.3)	3.2 (2.1–6.5)

### Relation FDG uptake and age of the prosthesis

**Figure 3.**
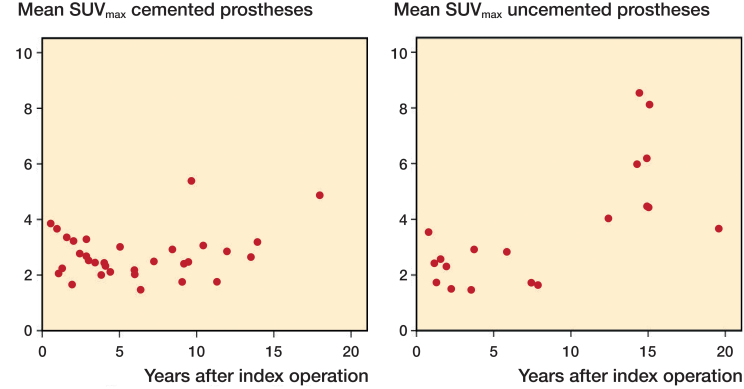
The relationship between the mean SUVmax and the age of the prosthesis in cemented (left panel) and uncemented hip prostheses (right panel).

The median time between the hip arthroplasty and the FDG-PET/CT scan was 3.9 years (IQR 1.2–9.1) in the cemented group and 6.6 years (IQR 1.6–15) in the uncemented group. In the cemented group, no relation was found between FDG uptake and the age of the prosthesis. However, a moderate relationship was found between FDG uptake and the age of the prosthesis in the uncemented group (Figure 3). This relation was observed for all 4 SUV variables (meanSUV_max_: *r_s_* = 0.63 [CI 0.26–0.84], p = 0.003; meanSUV_peak_: *r_s_* = 0.61 [CI 0.24–0.83], p = 0.004; meanSUV_max_/liver ratio: *r_s_* = 0.63 [CI 0.26–0.84], p = 0.003; meanSUV_peak_/liver ratio: *r_s_* = 0.65 [CI 0.29–0.85], p = 0.002). This was also observed in the results without correlated observations (meanSUV_max_: *r_s_* = 0.55, p = 0.04). The FDG uptake in the uncemented prostheses remains stable for the early years, but an increase was observed after approximately 13 years.

In general, the cemented total hip prostheses showed a mild decline in FDG uptake in the first 5 years after implantation (*r_s_* = –0.43, p = 0.03 [*r_s_* = –0.56, p = 0.05 without correlated observations]). After 5 years, the cemented prostheses show a relatively stable FDG uptake pattern over time.

## Discussion

The aim of our study was to determine the physiological FDG uptake in non-infected total hip prostheses. We found a diffuse heterogeneous uptake pattern in most prostheses with median SUVmax values of around 2.5, with the highest uptake observed around the neck. FDG uptake was positively related with the age of uncemented prostheses, but this finding was not observed in cemented prostheses. Our data could aid in the development of accurate interpretation criteria for FDG-PET/CT to diagnose a PJI.

An interesting finding was the increase of FDG uptake with time in the uncemented hip prostheses, which was observed after approximately 13 years after the primary implant. This FDG increase may be due to the development of wear particles (third particle disease) and/or subtle aseptic loosening, although no differences in implant survival between cemented and uncemented total hip prosthesis are described in the literature (Abdulkarim et al. 2013). It is important to note that the uncemented prostheses older than 13 years of age all comprised one brand of prosthesis: Biomet Mallory Head, a full-length hydroxyapatite-coated titanium stem combined with an uncemented titanium shelled cup. Therefore, we cannot rule out that the prosthesis design itself, instead of the age of the prosthesis, resulted in higher FDG uptake. Unfortunately, we do not have data from this type of prosthesis at earlier time points. Nevertheless, imaging interpreters should be aware that FDG uptake in non-infected older prostheses of this type may increase in time and may lead to false-positive results regarding diagnosis of a PJI.

Most studies that have been conducted so far on FDG uptake in hip prostheses evaluated visual uptake patterns associated with loosening or infection. In accordance with our findings, these studies concluded that physiological uptake is mostly seen around the neck of the prosthesis (Zhuang et al. 2002, Vanquickenborne et al. 2003), which theoretically may be explained by wear of components and an adverse tissue reaction. In addition, previous studies indicate that an infection is highly suspected if there is uptake at the femur–prosthesis interface. This finding is also in agreement with our data demonstrating that FDG uptake is almost absent in non-infected prostheses (Zhuang et al. 2001, Chacko et al. 2002, Manthey et al. 2002). Unlike previous studies, we also observed uptake at the lateral and medial acetabulum, which may be due to differences in the camera systems that were used. Previous studies used an FDG-PET without the addition of a CT scan. With the addition of a CT scan, higher resolution images are acquired and anatomical structures are better visualized.

Currently, the interpretation criteria of FDG-PET developed by Reinartz et al. (2005) is most often applied. The authors conducted a SUVmean analysis on the location with the highest uptake found around the prostheses. In that study, the SUVmean was 3.3 (SD 1.6) in prostheses without an infection, 5.0 (SD 3.0) in prostheses with aseptic loosening, and 5.9 (SD 2.7) in infected prostheses. With this analysis, the SUVmean between aseptic loosening and an infected prosthesis was similar. Unlike Reinartz et al. (2005), we performed a mean SUV analysis at 8 locations around the prosthesis, which may improve its diagnostic accuracy. Future studies should address the SUV values of these different locations in prostheses with aseptic loosening and infection.

This is the first study that compared the FDG uptake between cemented and uncemented hip prostheses and analyzed physiological FDG uptake over time. Furthermore, SUV analyses were performed at 8 different locations around the prostheses. With this number of locations, a more precise uptake around the prosthesis is noted than in previous studies and contributes to a better understanding of physiological FDG uptake. Future studies should focus on FDG uptake in septic and aseptic loosened total hip prostheses, to further develop adequate interpretation criteria for diagnosing PJI using FDG-PET/CT scan.

In summary, our findings provide insight into the physiological FDG uptake in non-infected total hip prostheses. In both cemented and uncemented total hip prostheses a diffuse uptake pattern is present with most uptake seen around the neck of the prostheses and no uptake in the soft tissues surrounding the prostheses. In addition, the level of FDG uptake in non-infected uncemented total hip prostheses is influenced by the age and probably the type of prosthesis. These findings may aid in the development of accurate interpretation criteria for FDG-PET/CT to better differentiate between (physiological) reactive inflammation due to a foreign body and a PJI.

SG: including patients, analysis of the data, interpretation and presentation of the results, main author. AG, PJ, and MW-B: study design, writing of the manuscript and supervising SG in analyzing and presenting the data. RB: analyzing the data and critical review of the manuscript. JP and GK: critical review of the manuscript. DVG: statistical analysis.

*Acta* thanks Zohar Keidar and Samantha Terry for help with peer review of this study.
